# Microfluidic Tools for Enhanced Characterization of Therapeutic Stem Cells and Prediction of Their Potential Antimicrobial Secretome

**DOI:** 10.3390/antibiotics10070750

**Published:** 2021-06-22

**Authors:** Pasquale Marrazzo, Valeria Pizzuti, Silvia Zia, Azzurra Sargenti, Daniele Gazzola, Barbara Roda, Laura Bonsi, Francesco Alviano

**Affiliations:** 1 Department of Experimental, Diagnostic and Specialty Medicine, University of Bologna, 40126 Bologna, Italy; valeria.pizzuti3@unibo.it (V.P.); laura.bonsi@unibo.it (L.B.); francesco.alviano@unibo.it (F.A.); 2 Stem Sel S.r.l., 40127 Bologna, Italy; silvia.zia@stemsel.it (S.Z.); barbara.roda@unibo.it (B.R.); 3 Cell Dynamics i.S.r.l., 40129 Bologna, Italy; azzurra.sargenti@celldynamics.it (A.S.); daniele.gazzola@celldynamics.it (D.G.); 4 Department of Chemistry “G. Ciamician”, University of Bologna, 40126 Bologna, Italy

**Keywords:** mesenchymal stem cells, stem cells, extracellular vesicles, exosomes, cell-therapy, anti-inflammatory, anti-bacterial, host-defense peptides, antimicrobial peptides, microfluidics

## Abstract

Antibiotic resistance is creating enormous attention on the development of new antibiotic-free therapy strategies for bacterial diseases. Mesenchymal stromal stem cells (MSCs) are the most promising candidates in current clinical trials and included in several cell-therapy protocols. Together with the well-known immunomodulatory and regenerative potential of the MSC secretome, these cells have shown direct and indirect anti-bacterial effects. However, the low reproducibility and standardization of MSCs from different sources are the current limitations prior to the purification of cell-free secreted antimicrobial peptides and exosomes. In order to improve MSC characterization, novel label-free functional tests, evaluating the biophysical properties of the cells, will be advantageous for their cell profiling, population sorting, and quality control. We discuss the potential of emerging microfluidic technologies providing new insights into density, shape, and size of live cells, starting from heterogeneous or 3D cultured samples. The prospective application of these technologies to studying MSC populations may contribute to developing new biopharmaceutical strategies with a view to naturally overcoming bacterial defense mechanisms.

## 1. Introduction

Today more than ever, the global population is aware of the impactful evolutionary abilities of the microbes wandering around our planet. Unfortunately, as with the microbiome, the pathogens have demonstrated an efficient molecular machinery that has allowed them to survive, evolve for centuries, and even turn into dangerous entities affecting human health. A compensatory response to the environmental changes [1] has led to mutations and greater skill in evading the human immune system [2], not to mention the development of multi-drug resistance (MDR). Antibiotic resistance has an increasing clinical and social impact, so both research on new drug candidates and antibiotic-free-based therapeutic strategies are expected to grow swiftly.

Biomimicry offers an alternative approach taking advantage of the natural sources of antimicrobial elements and learning from their chemical conformation and mechanisms of action [3]. Eukaryotes produce a variety of conserved bioactive agents, are able to protect themselves from pathogens, and some of this response can be emulated and translated as biomedicine against specific human pathogens. For example, many phytochemicals exert a broad range of antimicrobial activity and many natural compounds should be further characterized in new 3D physiological models [4,5] to show their anti-bacterial and immune-active potential. Like plants, animals possess natural molecules as part of their innate immune defense tools. The complement system plays a vital humoral role in such innate immune defense [6], which leads to opsonization by antibodies, recruitment of inflammatory cells, and direct attack on the pathogen [7]. Such an attack affects the cell membrane and can result in bacterial cell lysis [8] through the assembly of membrane-penetrating proteins called membrane attack complex (MAC). Besides complement and its role in preventing the invasion of the host tissues by the pathogens, the antimicrobial peptides (AMPs) are proven to have a certain bactericidal activity, while the endogenous cell populations engaged in homeostasis such as stem cells have a particular role during infection.

Stem cells are responsible for producing the mature cells of the organism. They participate in tissue homeostasis and cell renewal after birth. Mesenchymal stromal stem cells (MSCs) are somatic stem cells that repopulate the tissue of origin and display multipotency differentiation [9]. Their in vivo paracrine effects are critical for the resolution of tissue damage. Various stem cell populations can be isolated from perinatal tissues which are abundant sources of MSCs [10]. MSCs have been widely studied in translational medicine and frequently proposed as innovative drugs. MSC-based cell therapy involves the administration of MSCs to the receptor body, where they can move towards the affected tissues and contribute to healing. MSCs can transfer to the injured cells factors restraining injury and leading to tissue regeneration. MSCs can interact with the immune system and prevent infection via direct and indirect mechanisms [11]. In particular, the antimicrobial features are linked to paracrine release of several antimicrobial peptides (AMPs), with broad antibacterial properties, and specific extra cellular vesicles (EVs) secretion, including immunomodulatory factors. Depending on the origin of the tissue, MSCs differ functionally in their paracrine mechanisms and their secretome-derived products [12]. MSCs are considered medicines from a regulatory viewpoint [13], and hence their quality needs to be controlled like other medicinal products [14]. After only minimal manipulation, MSC administration to humans can be considered as a form of transplantation [14]. On the other hand, the ex vivo preconditioning has been proposed as a way of priming MSCs’ immunological features [15]. Efforts focusing on characterizing selective MSC features can now exploit the microfluidic technology, which proving to hold great advantages in the biomedical field.

While MSCs secrete their own EVs and AMPs, EVs protect and convey AMPs, and AMPs can also be directly MSC-secreted. MSC features include the detection of infection signals, the ability to migrate toward the infection site, and on-demand secretion of combinations of antimicrobial peptides (AMPs) [16]. The EVs released from MSCs retain antimicrobial characteristics [13] and are considered to be safer than parent cell administration [17]. The EVs as cell-free agents and/or drug carriers may have therapeutic functions in sepsis [18] and may evolve into superior drug delivery tools. The presence of AMPs in the cargo of EVs may represent the next cell-free therapy option against resistant bacterial infections. 

Microfluidic tools are being employed in cell factory and bioprocess development and can make new parameters accessible for evaluation [19]. The behaviors of cells are directly related to their mechanical environment. Microfluidic technology can enable formation of micron-scale gradients and single cell handling, as well as the analysis of cell behavior from single- to multi-cellular level. In cell biology, microfluidics makes it possible to combine mechanical or electrical stimuli with mechanical or electrical measurement factors [20]. In comparison with traditional cell culture conditions, microfluidic tools allow cell analysis in a more in vivo-like dynamic fluid situation. The idea of stem cells screening or selection by new microfluidic devices would be advantageous in reducing the number of candidates for future clinical application.

## 2. Host Innate Tools as Alternative to Antibiotics

### 2.1. Renewing Antimicrobial Peptides Potential

The AMPs, also known as host defense peptides (HDPs) are commonly found in multicellular eukaryotes, usually expressed constitutively [21] and, like the complement system, are evolutionarily ancient components. In comparison to antibiotics, AMPs may be considered “natural antibiotics” expressed across the phylogenetic kingdoms [22] and causing pathogen cell disruption through non-specific interactions with their membrane surface [23]. The majority of AMPs are cationic peptides that electrostatically interact with bacterial membranes or translocate into cells affecting intracellular targets. The first step of penetrating the polysaccharide surface consists in an electrostatic interaction between target bacterial membrane and AMPs, followed by attachment to the lipopolysaccharide, in Gram-negative, or to teichoic acid, in Gram-positive bacteria [2]. Afterwards, by creating pores in the membrane or perturbing its integrity with consequent function impairment, the AMP manages finally to kill the bacterial cell [24] followed by the leakage of intracellular components. AMPs are active towards both Gram-positive and Gram-negative bacteria. The positively charged component of AMPs is important for the selective interaction with the anionic bacterium membrane, whereas the hydrophobic part interacts with the hydrophobic bacterium inner-cell membrane [25]. The AMPs range from 5 to 50 cationic and hydrophobic residues and present an amphipathic conformation upon folding usually after interaction with membranes [26].

The existing repertoire of endogenous AMPs is an example of host-pathogen co-evolution, displaying smart adaptation to bacterial mechanisms of AMP resistance [27]. Among the AMPs expressed by a large number of species, which are divided on the secondary structure of AMPs, Cathelicidins and Defensins are the two main classes, and this is the case in humans as well. The protective role of these peptides is clear because down-regulation of them increases the susceptibility to bacterial diseases [28]. Cathelicidins, share the presence of a common cathelin domain and were first identified in bone marrow myeloid cells [29]; they are secreted as pro-peptides, mostly α-helical, by innate immune cells and include LL-37 (human Cathelicidin, antimicrobial peptide), which is perhaps the best known and uniquely human family member. Defensins comprise three subfamilies (α-, β- and θ) [30] of small (2–5 kDa) cationic peptides with a rigid [23] β-sheet core differently stabilized by cysteine linkage. Alternatively, depending on their final effect, AMPs can be separated into membrane disruptive AMPs and non-membrane disruptive AMPs [31]. In addition, during infection, hepcidin [32] reveals a defensive role through iron concentrations depletion while histatin shows a cytotoxic action similar to the candidacidal activity of human neutrophil defensin 1 (HNP-1) [23,33].

Thanks to their homology, the structure may result in an exogenous functional broad-spectrum possessing an anti-pathogenic effect. Significant evidence [34] is now challenging the simple and non-specific mode of action shared by AMPs [35], highlighting the influence of genetic variability of bacteria, and suggesting an additional role in regulation of the microbial symbiotic communities. Nonetheless, AMPs synergize each other, in presence of antibiotics and natural compounds [36]. In this context, AMPs can be used as rediscovered [37] immunological effectors, to damage specific bacteria and target lysis of the pathogenic surface. Recent reports include among advantages the lesser tendency [38] to generate resistance [39], a low propensity to develop toxicity [25], better control of infection by intracellular bacterial pathogens as opposed to antibiotic ineffectiveness [21], anti-biofilm effects [25,35,40], and activation of immune cells [41]. Interestingly the patterns of mobile genetic elements are entirely different between the antibiotic and antimicrobial peptide resistance genes [42].

Clinical implementation of AMPs is still a challenging area [28,43]. They are relatively safe due to their nature, but improved extraction and stability, low yield in recombinant heterologous expression [44], negligible toxicity expectantly in organoids and other 3D cellular models [45], and lowering the costs needs to be assessed, including the engineered synthetic production as inspired by nature. Only a few AMPs are studied in ongoing clinical trials [46], but they are currently seen as promising candidates and future alternative to conventional antibiotics [47]. In addition, AMPs are pleiotropic molecules [48] that display immunomodulatory properties [23,45,49] and induce cytokine production, immune cell homing, and differentiation [50].

Nanomaterial research [41,51] and seeking sources of AMPs as well as other immunomodulatory agents are a promising avenue for reducing antimicrobial resistance [52] and combating infectious disease [25,37]. Due to low permeability due their molecular size, non-specificity, and quick degradation by enzymes in body fluids, the risk of a systemic toxicity is high so optimization of the delivery system [22] will be a critical step in enhancing AMP in vivo bioavailability. The prospect of employing AMPs directly from their natural sources is one of the emerging suggestions of this review.

### 2.2. MSCs as Source of Antibiotic-Free Nanomaterials

In regenerative medicine applications mesenchymal/stromal stem cells (MSCs) have acquired a leading position [53]. Indeed, tissue engineering is becoming the most important biotechnological clinical application requiring biomaterials and transplantable cells such as MSCs. Such somatic stem cells can be isolated from many organs and have shown a well-documented multipotent differentiation potential toward tissue that needs to be replaced after damage or degeneration. Bone marrow was the first source of MSCs but adipose tissue is currently one of the most used. In addition, perinatal derivatives [10] like the placental membranes [54,55] and the umbilical cord are considered clinical waste material, and together with the amniotic fluid are great resources [56] for the isolation of immunotolerant and immunomodulating [57] stem cells, including MSCs [58]. MSC isolation does not raise ethical issues; they are non-tumorigenic in vivo and are a well-tolerated medicinal tool suitable [59] for cell therapy purposes. MSCs have demonstrated success in clinical areas such as hematology, orthopedics, and reconstructive surgery and are now identified as a future key player in various therapeutic areas such as diabetes, cardiovascular and degenerative diseases. Recently, clinical evidence of the MSCs’ usefulness against infectious diseases was borne out by the number of active clinical trials involving MSCs from different origins as a treatment for COVID-19 related pulmonary disease [60,61,62,63]. This has led to current pre-clinical investigation of the optimal source yielding the best anti-inflammatory profile [64], such as MSCs from placenta [65] and their paracrine activity [66] through exosomes [67]. In such a context, we have seen the importance of MSC manufacturing and controlling their quality, which may reflect the cell behavior and biological effect of the secretome [68].

Although cell reconstitution is an essential component of MSC-based tissue engineering application, the therapeutic [68] and protective effect of MSCs is now mainly thought to derive from their secreted factors enhancing cell survival [69] and from intrinsic immunomodulatory functions [70]. MSCs possess immunomodulatory [71,72] and immunosuppressive [73] properties, and thanks to the low expression of the major histocompatibility complex (MHC) class I and almost absent MHC II, they are considered immune privileged cells upon isolation, a state that could be progressively lost during in vitro cell maintenance. MSCs interact with the innate immune system in the antimicrobial responses; they influence macrophage polarization by inducing M2 phenotype, as well as attracting and activating neutrophils [74].

Accumulated evidence suggests that the therapeutic benefit of MSCs belongs mainly to their paracrine action [75,76], rather than to MSC differentiation after transplantation. Indeed, the current literature supports the notion that MSCs effectively act via their secretome [77] in several clinical conditions [78]. Notably, the expression of indoleamine 2,3-dioxygenase-1 (IDO-1) [79], Human Leukocyte Antigen-G (HLA-G) soluble molecules, interleukin-10 (IL-10), and prostaglandin E2 (PGE2), which are characteristic and efficient immunomodulatory molecules secreted in large amounts by MSCs [13]. It is important to preserve MSCs from excessive stress and alteration deriving from in vitro expansion, in order to maintain the curative and adjuvant properties connected to their secretome. Together with culture optimization, preconditioning of MSCs with exogenous stimuli, such as chemical (small molecules, natural antioxidants, synthetic drugs), physical (hypoxia, biomaterial contacting), or biological factors (chemokines, trophic factors, toxins, AMPs) has been proposed as a strategy to improve MSCs activities in vitro and in vivo [80] and enhance their therapeutic effect [81].

MSC–based therapy is under consideration for sepsis disease [82]. The challenge is to identify the organ of origin with the highest availability, abundance and with the best-expected effects in terms of anti-infection strategy [83]. Significantly, MSCs have been indirectly associated with the attenuation of virulence or clearance of invading pathogens [84]; for instance, MSCs enhanced host survival and bacterial clearance in a murine model of pneumonia [85]. A series of studies led to the current hypothesis that MSC treatment may in future be an effective strategy for severe pneumonia [86]. Thus far, antibacterial effects from MSCs have been reported [11,12,13,84,87,88,89,90,91], for instance their role in bacterial clearance of MSCs-derived AMPs that directly boosts the innate immune response [16]. AMPs and MSCs share the ability to fight infections through direct microbicidal properties and/or by modulating the immune responses. Currently MSCs have been shown to constitutively express the following AMPs [16]: cathelicidin LL-37, human β-defensin-2 (hBD-2), hepcidin, and lipocalin-2 (Lcn2) [85]. Among the first AMPs studied [92] and the ones that this review considers, antimicrobial effects have been attributed to the LL-37 function. It remains to be explored whether MSC-released vesicles conserve antimicrobial activity by their AMPs content or not. In the case of epithelial cells of the urinary tract expressing the AMP dermcidin [93] or from the biliary and intestinal epithelium carrying LL-37 and hBD-2 [94], the secretion of AMPs may be conducted by MSCs through extracellular vesicles (EVs). Another example is the administration of vesicles secreted by bone marrow MSCs (BM-MSCs), which reduced the influx of bacteria and inflammatory cells and improved survival in a bacterial pneumonia mouse model. This finding also demonstrates that the MSC-vesicles have the same protective and immunoregulatory effects as their parent cells [95]. Moreover, conditioned medium (CM) from adipose derived MSCs (ASCs) has shown exciting results in this connection. ASCs expressed LL-37 at the mRNA level [96], and their CM inhibited the growth of *S. aureus*, while in another study CM, in synergy with the antibiotic geneticin, decreased the *P. aeruginosa* growth rate [16].

Mastitis is characterized by infection with the pathogen *S. aureus,* usually treated with intramammary antibiotics. Encouraging reports have been recorded in the veterinary field, mainly in the treatment of mastitis models using CM from bovine bone marrow and adipose tissue MSCs [97]. In another study, CM treatment led to a lower rate of relapses than with antibiotic treatment [98].

Again, canine BM-MSC-CM has shown in vitro activity against *S. aureus* biofilm and quorum sensing in Gram-negative bacteria [99]. A solid recent in vitro and in vivo study [87] reported that administration of MSCs as coadjuvant to a conventional class of antibiotics, exerted a direct and indirect antimicrobial effect in a *S. aureus* mouse infection. It was found that the combination of colistin antibiotic with MSCs improved the treatment of *K. Pneumoniae* infection in a neutropenic mouse model [100]. Yoshitani et al. recently found that the local administration of ASCs combined with ciprofloxacin antibiotic decreased abscess formation and the bacterial burden in implant-associated osteomyelitis infection by *S. aureus* [89]. Human umbilical cord blood MSCs were tolerated in septic mice, fewer neutrophils were recruited to the liver and the MSCs mitigated the severity of LPS-induced sepsis [89]. Similarly to BM-MSCs expressing CD362 (i.e., syndecan-2) [101] that were effective attenuating *E. coli*–induced injury, the cells isolated from umbilical cord tissue and expressing the same marker had additional effects comparable to antibiotic therapy and rescued the mice from *E. coli* injury following bacteria instillation [102].

The antibacterial properties of MSCs are probably influenced by their origin. It is suggested that Wharton Jelly Umbilical matrix (WJ-MSCs) [103] may be preferable in sepsis modeling [104]. In vitro phenomena such as phagocytosis, secretion of oxygen free radicals, and antibacterial molecules were reported for ASCs when placed in contact with a large panel of both Gram-negative and Gram-positive bacteria, whereas in vivo they reduced the bacterial load of murine periopathogens [105]. The amniotic membrane homogenate has antibacterial properties, and the amniotic membrane has the potential to be used against multidrug-resistant bacteria [106]. Treatment with IL-1β of amniotic membrane, a well-known source of perinatal stem cells [107,108], resulted in an augmented secretion of AMPs including hBDs and LL-37 [109], which is important when it comes to using the amniotic membrane as an antimicrobial scaffold in regenerative medicine.

The indirect antimicrobial effects of MSCs, partly mediated via Toll-like receptor (TLR) signaling [110], proved to (I) modulate proinflammatory cytokine and chemokine induction; (II) to release immunosuppressive factors that inhibit excessive proliferation and infiltration of inflammatory T cells and Natural Killer (NK) cells; and (III) to increase phagocytic activity of monocytes and neutrophils [111]. In addition to the inhibition of the NLRP3 inflammasome [112] by BM-MSCs [113], the ASCs [113] also reduced the activation of NLRC4 inflammasome [114,115], thereby increasing the phagocytic ability of macrophages induced by *P. aeruginosa* infection [116]. Treatment with MSCs diminished the nuclear factor kappa-light-chain-enhancer of activated B cells (NF-kb) in lung injury induced by sepsis [117]. This pathway is linked to IL-1β and Toll-like receptor (TLR) signaling. In recent years, IL-1β and a Toll-like receptor (TLR) agonist [118] were investigated as biological factors by which to prime MSCs [118,119]. Augmented T-regulatory cell induction is a consequence of MSC-induced TLR-activation [120], while the preconditioning approach in an inflammatory milieu could facilitate the appearance of MSCs with anti-inflammatory properties [121]. LPS preconditioning of MSCs modulated the immune response and reduced inflammation after transplantation into septic mice [122]. The stimulation of MSCs with TNF-α and IFN-γ led to the release of EVs with enhanced anti-inflammatory properties [123]. On the other hand, MSC-EVs can induce macrophagic phagocytosis [124].

To sum up, there is growing evidence that, as part of the secretome, the extracellular vesicles (EVs) will be the next effective therapeutic agents deriving from MSCs. Current research concerning MSC-EVs [125,126] as anti-inflammatory [127] and pro-regenerative agents for treating inflammation-related conditions has shown therapeutic potential in pre-clinical studies as recently reviewed [128]. In most of the cases persistent infections led to increased tissue damage associated with excessive duration of inflammation. Therefore, a therapeutic approach combined with the antimicrobial, anti-inflammatory and regenerative effect of stem cells would seem to be ideal in chronic infections.

## 3. Antibacterial Exosomes as Future Biomedicines

The definition of extracellular vesicles (EVs) involves multiple biological meanings. Basically, they are subcellular components secreted by a paracellular mechanism, a heterogeneous group of spherical lipid double-layered nanostructures. The majority of the studies in the literature define the diameter of exosomes as ranging from 40 nm up to 150–200 nm, whereas the size of microvesicles (MVs) typically ranges from 100 nm up to 1 μm [129]. The EVs are recognized as significant mediators of intercellular communication that enable inter-kingdom crosstalk, considering environmental Darwinian competition. Thanks to the multiplicity of transferred molecular cargoes, EVs offer a simultaneous delivery of various messengers to local or remote sites [130]. Note that they have been implicated in many physiological cell activities such as stress response, gene transfer (via RNA or DNA), delivery of virulence factors, pathogenicity, detoxification, and modulation of the host immune response [131].

The shedding of microbial extracellular vesicles constitutes a universal conserved mechanism for inter-kingdom and intra-kingdom (trans-kingdom exchange of biomaterials) communication [132] and can manipulate host immune response [133]. Successful application in the biomedical field of outer membrane vesicles (OMVs), naturally secreted by Gram-negative bacteria, has led to them being proposed as the basis for a promising antigen delivery system and being found in ongoing vaccine development [134]. The advantage for a vaccine platform stems from OMV’s built-in adjuvanticity [135] and size-dependent ability to induce both humoral and cell-mediated immune responses as well as from the delivery of heterologous antigens by engineered OMVs [136] in a natural conformation [137].

There has been speculation about the role of EVs in mediating cell protection [138] and the host response to infection [128]. Recently, it was also reported that human EVs and exosomes act as cellular decoys and are produced by cells for protection against bacterial pathogens [139,140].

Recently, the study of exosomes moved from their pathophysiological role to a therapeutic use based on stem cells. The EVs can be innate biotherapeutics, while cellular preconditioning is a promising method of advancing the production of therapeutic EVs [141]. The exosomes have been reported to be stable and to resist degradation in biological fluids, protecting their content attaining the target [142]. Generally, since EVs display low immunogenicity, are non-mutagenic [141], and have great physiochemical stability, they will be promising protagonists of future nanomedicine [141]. Considering the therapeutic effects exerted by MSCs, the development of an EV-based approach aspired to translate their anti-inflammatory agents into a future nanosized treatment for inflammation-related conditions [128].

EV purification from stem/progenitor cells most likely reflects the parental cell phenotype, thus inhibiting or enhancing the immune response [143] as well as influencing infection and inflammation levels. Exosomes and microvesicles belonging to MSCs, collectively known as MSC- extracellular vesicles (MSC-EV), have overlapping size ranges [76], so the various methods employed to sort them have led to different results and non-rigorous classification of the vesicles collected. In the last decade, the specific properties and function of MSCs exosomes [144] has gained great appeal [145]. Especially EVs obtained from MSCs seem to possess the advantage of having an intrinsic regenerative and immunomodulatory potential [76,146]. Exosomes secreted by MSCs contain consistent immunomodulatory mediators, including growth factors such as transforming growth factor-β (TGF-β) and hepatic growth factor (HGF), anti-inflammatory chemokines such as IL-10 and IL-1 receptor antagonist (IL-1Ra), as well as the typical aforementioned MSC factors, i.e., IDO-1 and PGE2 [76]. The MSC-EVs have been shown to influence the balance in macrophage polarization, in particular by promoting the switch from M1 to M2 phenotype [147,148]. Stimulation of cells with interferon gamma (IFN-γ) and tumor necrosis factor alpha (TNF-α), caused MSC-EVs to be released [149]. Placenta MSCs-derived exosomes show protective effect against senescence [150] while BM-derived exosomes have been identified as senotherapeutics [151,152]. Among further advantages of MSC-EVs, first there is their crossing of biological barriers, from the easiest plasmatic membrane to the hardest blood–brain barrier [153], secondly their ease of preservation and storage. MSC exosomes represent a sub-group of EVs that is present in the conditioned medium (CM) of MSCs. As mentioned, sizes overlap between MVs and exosomes is overlapping, making distinction and separation still unsolved issues. However, the components of CM can be separated by centrifugation, filtration, polymer precipitation-based methodologies, ion exchange chromatography, and size-exclusion chromatography. Rapid advancements of microfluidic and lab-on-a-chip technologies will enable EV isolation [154], purification and integration of physical and biochemical analyses [155] for the identification of relevant MSC-EVs subpopulations. The starting material for purification of EVs may be biological fluids such as plasma, urine and saliva, or cell culture medium for in vitro expanded stem cells. Hence, to pave the way for an EV-based clinical approach, there is a current need to improve the quantitative and qualitative methods for EV production and standardization [77,156].

As a result, the combination of antibiotics, that usually interfere with key events in pathogen replication, with bactericidal agents (e.g., AMPs), that directly attack bacterial structures such as the membrane, and finally with immunomodulatory agents, comprised in distinctive patterns of MSC-derived EVs, emerges as the leading strategy to counteract chronic and severe bacterial infections. Owing to the immunomodulatory and anti-inflammatory capacity of MSCs [127], the exosomes secreted by these cells given certain physiological conditions or specific priming (preconditioning) could be enlisted as complementary anti-bacterial agents, in substitution for or combination with antibiotics. EVs deriving from MSCs, conditioned with Toll-like receptor TLR3 agonist, showed enhanced antimicrobial effect compared with those from unconditioned MSCs [141]. Similar results were obtained by Park et al. who observed increased antimicrobial activity by MSC-MVs in an ex vivo perfused human lung suffering from bacterial pneumonia [157]. LPS stimulation of MSC-EVs inhibited the LPS-dependent NF-κB pathway, thereafter, regulating macrophage plasticity [158]. IL-1β-conditioned MSCs had EVs capable of attenuating murine sepsis by evoking an anti-inflammatory M2 response [159]. The latest insights suggest that CD14 is associated with the molecular signature of antimicrobial MSC type and the overexpression of CD14 can create a condition responding better to future bacterial challenge [160]. After conditioning of BM MSCs with staphylococcal enterotoxin B, the antibacterial peptides Hepcidin and LL-37 and anti-inflammatory cytokines proved up-regulated, *E. coli* growth in vitro has been reduced more than naive CM, while in vivo there was an improved bacterial clearance was found in septic mice [161].

We would like to highlight the prospective potential of MSC-derived exosomes [75] as a drug carrier [162] with their endogenous capacity for immune system modulation, as they contact different cell types, by minimizing the proteolytic degradation and avoiding the limitation of pure preparations of AMPs [37]. Thus, MSC-exosomes may counteract microbial pathogenesis in a more physiological way. Despite the advantages of liposomes in therapeutic delivery, there are many hurdles, for example their rapid clearance. Importantly, EVs are natural nano-sized carriers [138], though holding greater potential than liposomes in the drug delivery research areas [163]. In comparison to the liposomes, which usually elicit complement system activation, EVs also present an endogenous loading according to the specific source of their biogenesis. MSC-exosomes could hold the homing ability of native MSCs toward an inflammation site [164]. Their natural origin will confer a biocompatible profile while the specific protein pattern on the surface of MSC-exosomes will favor innate organotropic homing [165]. Being nanomaterials, the exosomes from MSCs can be delivered by biomaterials, promoting broader therapeutic effects in regeneration approaches or in vivo controlled delivery. Ongoing clinical trials are assessing the administration of MSC-exosomes in COVID-19 patients [166].

To sum up, the EVs, especially exosomes, can serve as carriers of nature-inspired synthetic antimicrobial peptides, hopefully enhancing the therapeutic effects and limiting undesirable side effects. EVs obtained from cultured MSCs will add an immunomodulatory and regenerative therapeutic potential.

## 4. Microfluidic Tools for Biophysical Selection of MSCs

To develop proper and safe new MSC-based therapeutic strategies against infectious disease and sepsis, the choice of source together with a well-characterized MSC population need to be addressed. In addition, the use of cellular products such as extracellular vesicles (EVs) as a therapeutic alternative still requires extensive study. Technological approaches to improve the physiological testing conditions facilitate innovative characterization of MSCs, thus contributing the requested refinement of the quality control of the starting material used to produce the cell-free therapeutic material [167]. Microfluidics analysis can investigate cell phenotypes whilst maintaining their physiological conditions. It is an expanding field that has already led to successful development of point-of-care diagnostic portable systems and platforms, lab-on-a-chip for drug discovery and screening, basic life science research tools, and sophisticated analytical devices. In the future, microfluidic technology seems likely to make significantly advances in the production and quality control of size-controlled EVs as promising drug delivery systems [168]; it will be used in a greater number of microbial studies and in combating infectious diseases [169].

The use of fluidics in MSC research is not new: cells and in particular MSCs are screened by flow cytometers or selected by a fluorescence-activated cell sorter (FACS) following their predictive surface antigen expression pattern [170]. To define an MSC, cells need to positively express putative mesenchymal markers and be negative for the hematopoietic ones. However, no specific panel of markers exists by which to identify the ideal MSCs because many are co-expressed by different types of cells. In addition, antibodies add significant costs to cell production and set back process of the translating sorting techniques to clinical settings [171]. MSC exosomes express their parental cell markers [172] and could be enriched by immunomodulatory components, such as TGF-β [173]. Like MSCs, the exosomes have a characteristic pattern of tetraspannin proteins expressed on their lipid layer, such as CD9, CD63, and CD81 [174]; antibody detection enables a sensitive identification of exosomes suitable for enrichment and excluding phenotypically different contaminants. Immuno-tags are also working in parallel with size-exclusion of apoptotic bodies (vesicles) or unwanted types of Evs. In the hope of increasing high throughput and sensitivity, microfluidics-based technologies have been gradually employed in strategies for the isolation and analysis of Evs [154]. However, some label-free / tag-less methods are needed for separating and isolating cells possessing distinctive and desired properties which are essential for the clinical translation. Many biochemical techniques exploit the physical properties of the cells, such as sedimentation, precipitation, and flocculation, which are the best known. Today, interest has revived in the physical properties thanks to new methods focusing on the biomechanical aspect of tissue engineering, the stem cell niche remodeling and the extracellular matrix signaling. The cellular microenvironment delineates the interacting player in the physical forces that govern cells state and behavior. It is fundamental to remember that the sum of the physical characteristics correlates to a distinct phenotype, providing a signature for the different therapeutic activities. The differences may depend on the unique features of the stem cell sources and the variation in their biochemical content; for this reason, the current aim is to find a combination of biophysical and morphological properties such as to reveal an advantageous phenotype. This would be enticing not only for primary single-cell suspension, immediately after tissue isolation, but also for cells growing in physiological-like 3D-culture [175], such as spheroids, which have proved indisputably these last few years to mirroring of the in vivo conditions.

Some emerging microfluidics-based technologies show the potential for a biophysical characterization of stem cells and their further selection thereof. Devices incorporating different fluidic technologies all tend to measure the biophysical cell parameters. The conventional methods of studying cell heterogeneity, such as flow cytometry or immunofluorescence microscopy require cell manipulation or even cell fixation. In the most evident example of physical parameter analysis, forward versus side scatter, using the flow cytometer does not yield information about the cell state and physical details. On the other hand, technologies based on fluidics [176] have allowed the measurement of morphologically distinctive patterns and physical characteristics but these have not yet been defined yet as a signal of cellular processes. Cell density, which is usually not analyzed, may be included among such underrated characteristics. Sedimentation is not useful for rare populations like stem cells and is difficult to control. The purity of density gradient centrifugation is low and the knowledge of the density of target cell type is a requirement [177]. Cell density correlates with cellular content, especially the cell protein fraction, while in multicellular objects it reflects the complexity of the intercellular junctions and the extracellular matrix. Density and other biometric factors, such as the surface curvature and rigidity, bear a strict relation to the force of gravity. By exploiting this universal force, heterogeneous cellular samples, including stem cell populations, can be distinguished and selected, with the support of a couple of innovative microfluidic devices.

Celector© technology is based on the field flow fractionation (FFF) assisted by the earth’s gravity which works as a separating external field [178]. This device, designed specifically for cell separation, can be defined as a cellular chromatograph. The cells from raw ex vivo samples can be analyzed and separated in an isotonic fluid, meanwhile performing non-invasive and single-cell multi-biometric quantification. Label-free cell characterization avoids signaling cascade activation, thus preserving the stemness in native cells. Cells possessing different physical characteristics may express a different secretoma that could be encapsulated in the EV formation. The instrument has proven to be able to identify and sort sub-populations of amniotic fluid stem cells, while the cells collected from each fraction show a different transcriptome expression profile [179]. It will be interesting to assign a specific fingerprint for each tissue source and investigate the MSCs biophysical parameters of the fraction better expressing antimicrobial features. Through such technology, it would be possible to compare a known MSC anti-microbial fingerprint with new cell samples, deriving from the same donor but from later passages. Hence, the added value of the instrument is the possibility firstly to have a quality control check on the state of the cells, identifying the right subpopulation from complex or even expanded cells, and subsequently to isolate their secretoma. Then, after demonstrating the best antimicrobial effect in downstream analysis, there comes the enrichment and subsequent expansion of such anti-microbial cell phenotypes. Microfluidic separation may have the potential to check the success rate of priming strategies on MSCs, for anti-microbial and anti-inflammatory purposes. In addition, monitoring after conditioning with microbial substances or cytokines may unravel differential binding toward different tissue sources. The MSCs can be exposed to bacterial virulence factors and processed by Celector^®^ in order to detect resistant cell fractions endowed with the potential to counteract the infection. Dead cells and debris have shorter retention times than the other cells, eluting in the first minute of the analysis, so that the likely healthy fractions can be detected and collection of them guaranteed. In summary, there will be many opportunities to use label-free field flow fractionation of MSCs in regenerative medicine, cell therapy and infection disease research.

From another angle, there is growing consent demands for the integration of microfluidic tools in 3D cell cultures. Both organoids and spheroids can be formed by primary cells and stem cells. The combination of these 3D cell cultures with microfluidic systems, such as a sophisticated organ-on-a-chip [180] will develop more and more relevant in vitro pre-clinical models [181] to study human pathology, accelerate discovery and screening of new drugs, or biologics such as new host defense peptides [45], and evaluate treatments. The limitation of flow cytometric analysis is the inevitable disruption of spatial spheroid organization by cell dissociation and loss of their whole properties as unique multicellular elements. It seems that scientists still lack methods for physical cytometry and standardization of 3D cellular heterogeneous models. The presence of automated sample tracking would be an advantage as well as a requisite for high-throughput technology. Secretion of trophic factors is favored in 3D cell culture. By establishing spheroid culture, more cells can be obtained than by monolayer cultures [182], therefore secreted factors amount increases together with an enhancement in anti-inflammatory factors production [183]. In stem cell spheroids the relative hypoxia located at the center of the sphere has a critical role [184] in the maintenance of stemness and for clinical application of MSCs [185]. Protein synthesis and cell volume increase can be uncoupled [186]. Minimal changes in single cell density suggest variations in cellular processes and contents that are hard to detect by mass or volume measurements. For example, cell senescence is accompanied by changes in cell density [186]. It can be conjectured that other cellular processes important for stem cell functions will likewise affect the cell density value. Indeed, red blood cells infected by *P. falciparum* and non-infected healthy cells prove distinguishable by comparing the distribution of their density instead of their mass [187].

In the past, one method to measure single-cell density has involved a dual suspended microchannel resonator device [188]. Yet, the density of a cell aggregate is a quantitative indicator of cells compacting themselves. The degree of compactness of cell aggregates like spheroids will reveal the collective variations in the single cells comprised in them, which are connected to growth and cell cycle changes, modification of the inner cellular composition, but will also redirect to the number of cells and their intercellular connection network.

Within the panorama of microfluidic devices for studying 3D models, we should mention the fluidic network of the W8© instrument, where single spheroids fall freely in a specifically conceived analysis flow-channel dedicated to analyzing their terminal velocity [189]. Thanks to this analysis the values of weight, size, and, importantly, mass density of the 3D biological sample are measured. Mass density can be a valuable indicator of spheroid state and could be useful in following up samples with specific properties or distinguishing between spheroid populations that have very similar mass or volume. Thus, the monitoring of MSC spheroids would benefit by such density analysis so as to maintain the quality of the antimicrobial or immunomodulatory populations, which would be instantly characterized upon isolation and even expanded in vitro in 3D culture conditions. In addition, such technology could be useful in checking the phenotype of pre-conditioned MSC cultures forming significant anti-microbial and immunomodulatory spheroids [190], thus encouraging standardization of 3D cell cultures [191] for future clinical application. The loss of density in a spheroid phenotype derives from damage at the intercellular level and cell-matrix level as well as alteration of junctional complexes. Since different biological factors, such as toxins and inflammatory chemokines, could have long-term unexpected consequences for the spheroid state, the density would be a valuable parameter to ascertain for early control of the optimal conditions needed for efficient MSC-priming without affecting cell health. Considering the organoid-based insights and promise of organoids as a tool advancing SARS-CoV-2 research [192] recent findings of MSC-based clinical trials designed for COVID-19 treatment [60], we have a great opportunity to understand the potential of this technology in characterizing MSC spheroids and profiling them for anti-microbial quality ranking.

**Figure 1:**
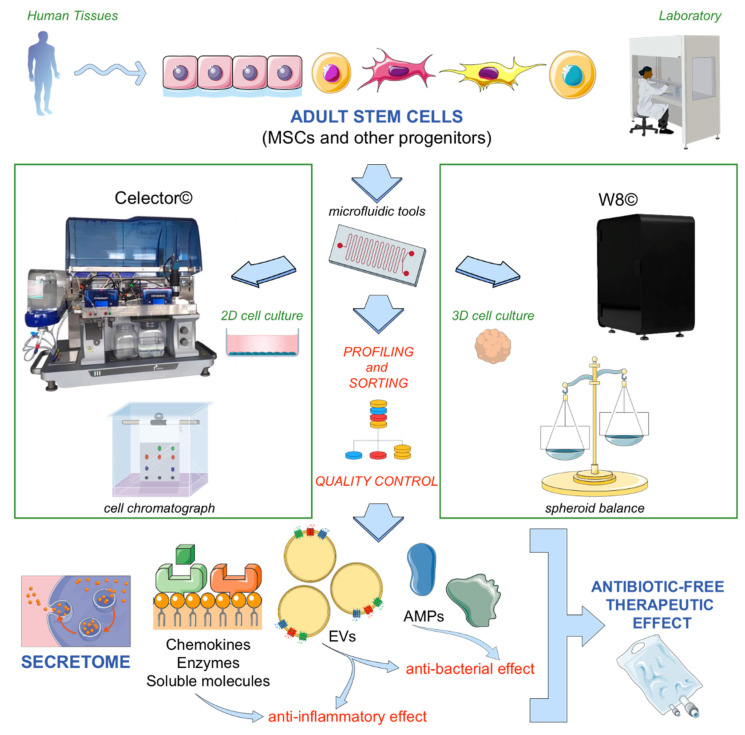
Biophysical characterization of stem cells for the prospective prediction of their secretome by selected microfluidic devices.

As both a technical limitation and an operative advantage, the above-described microfluidic-based technologies need their own imaging-aided software. Importantly, they both enable the sorting of biological samples and in this manner can provide some new label-free approaches [193] for the characterization of stem cells at a higher physiological grade. As Figure 1 schematically shows, Celector© and W8© technologies are cooperative fluidic platforms that can be applied to both 2D and 3D cell culture conditions. They may represent tools to improve the profiling and quality control of stem cells (Figure 1), before to proceed with any subsequent antibiotic-free products manufacturing.

## 5. Concluding Remarks

By 2050 the global antimicrobial resistance threat will lead to more deaths from bacterial infections than cancer [194]. New approaches for the prevention of implant-related resistant infection will involve elective transplantable cells having “medicinal effect”, such as MSCs [195]. The current expansion of multidrug-resistant pathogens has highlighted the demand for host-protecting molecules, and one of the candidates for novel anti-infective therapeutics is the AMP [46]. As these molecules possess a wide range of bacterial susceptibility and complementary immunomodulatory functions beyond microbicidal activity [33], AMPs and their native container vesicles will rapidly gain attention thanks to their emerging clinical potential. Moreover, MSCs and their microvesicles/exosomes have proven their immunomodulatory and anti-bactericidal ability in many preclinical studies. The innate ability of MSC derived exosomes to deliver their cargo to the damaged tissue forms the basis of their innovative application as a drug delivery system in infection therapy. Pathogenic bacterium damage could indeed be targeted by natural delivering nanomaterials [51], for instance by the combination of mammalian microvesicles and exosomes coupled with selected natural AMPs or synthetically optimized peptidomimetics [54,196,197], in substitution for or synergy with classical antibiotics.

Nevertheless, optimization of a homogeneous cell preparation from which to derive therapeutic cell-free components, without altering the cell phenotype and characteristics, is still a challenging issue. By way of support for better characterization of MSCs—cells capable of intriguing antibacterial effects [84]—novel cytometric technologies can signally contribute to meeting our urgent need for antibiotic-free therapies (Figure 1).

In conclusion, MSCs and their secretome hold strong potential in the quest for alternative/adjunctive therapies to MDR bacteria in combination with antibiotic solution [198,199] and biofilm [111], which are the reference therapeutic approaches. In this perspective, new methods of biologic quality assessment [200] will be an essential step prior to the clinical use and for exploring the role of EVs as anti-microbial agents.

## Data Availability

Data is contained within the article.
